# Supplementary material for: ACE2 protein expression within isogenic cell lines is heterogeneous and associated with distinct transcriptomes
Source: Sci Rep. 2021 Aug 5;11:15900. doi: 10.1038/s41598-021-95308-9 (PMC8342525; doi:10.1038/s41598-021-95308-9)
Supplement: Supplementary file 1 — Supplementary Figures. [file 41598_2021_95308_MOESM1_ESM.pdf]

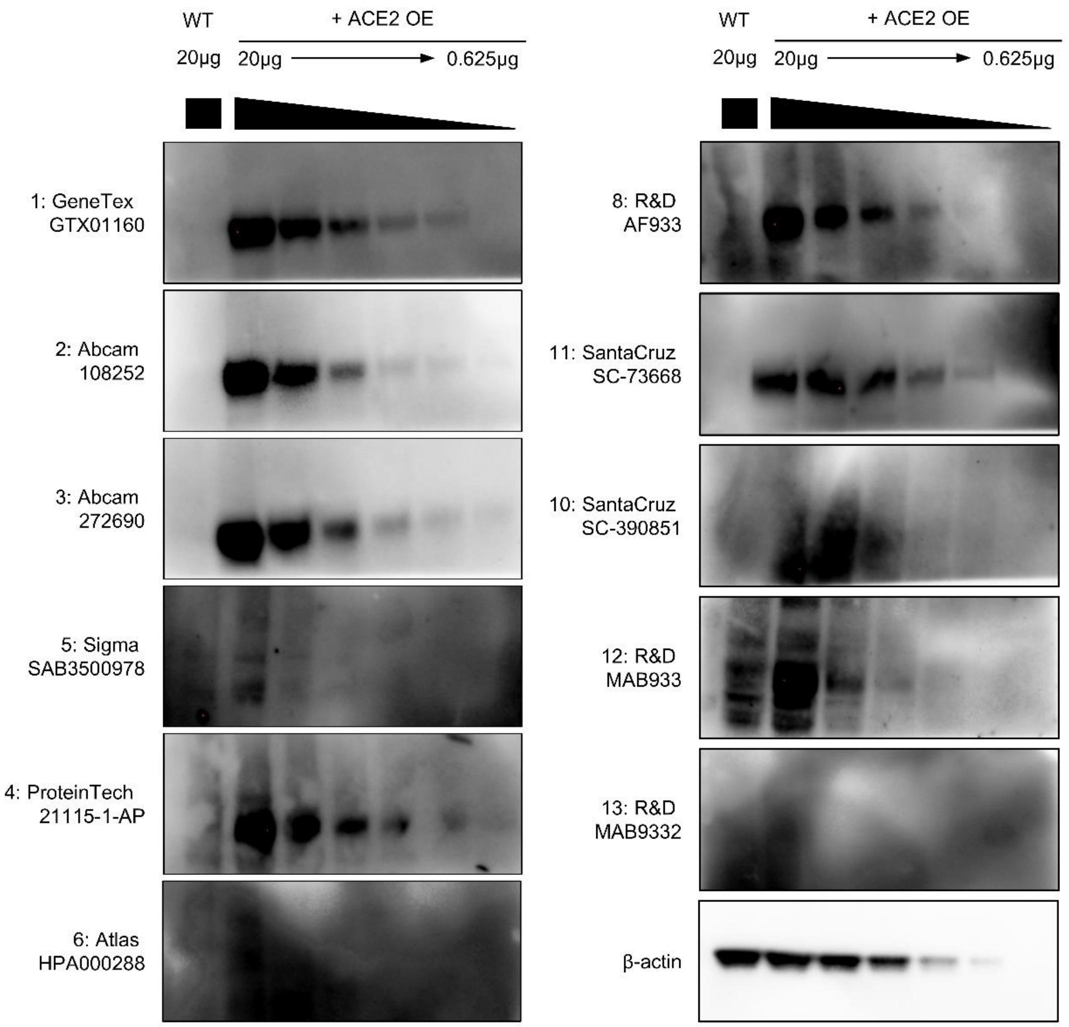

Supplemental Figure 1. Assessment of ACE2 antibody sensitivity and specificity by immunoblotting. Representative immunoblots for ACE2 using each of 11 different commercial antibodies, comparing 20 μg of lysate of HEK293T parental cells to a dilution gradient of 20 μg to 0.625μg of lysates from ACE2-overexpressing HEK293T cells.

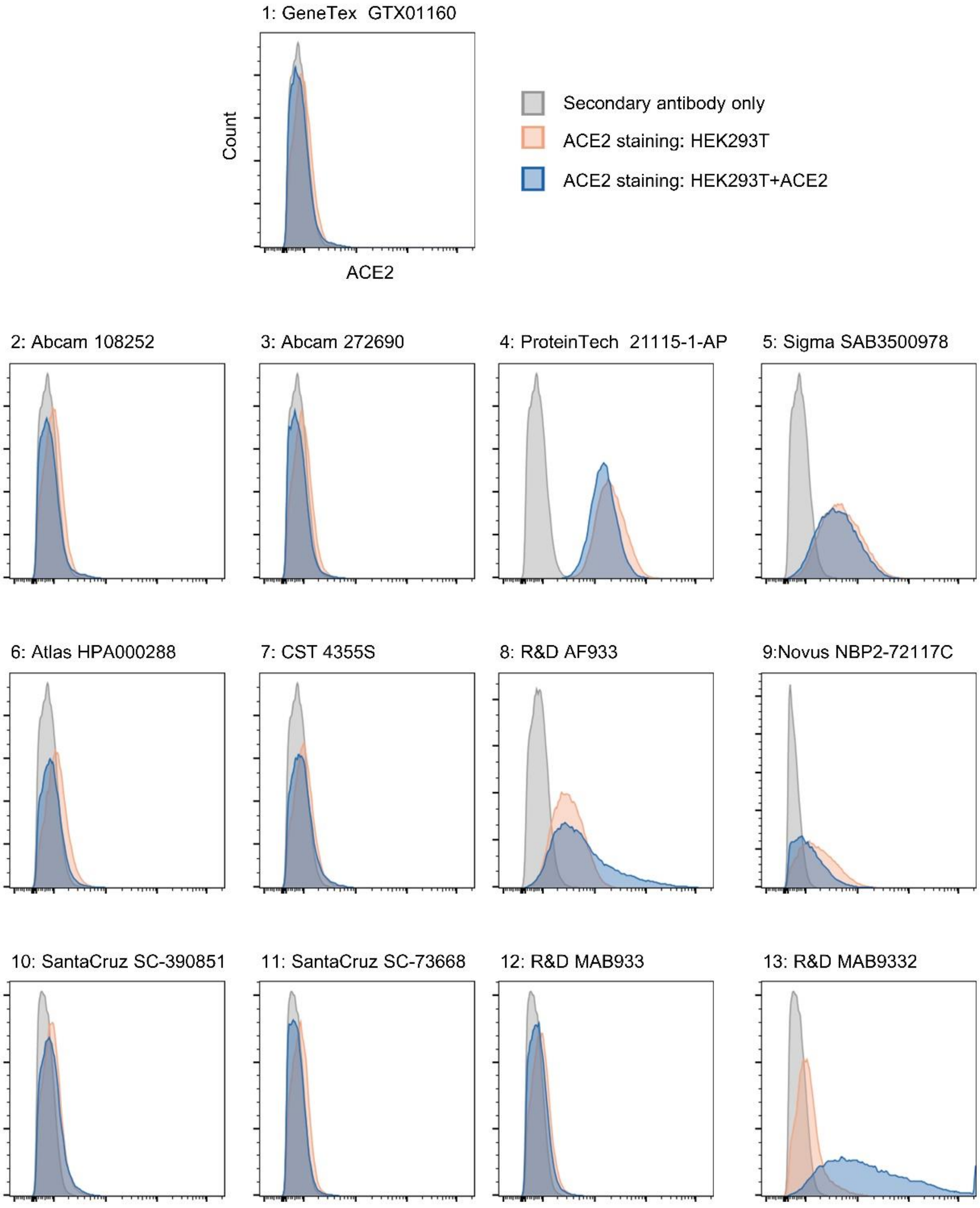

Supplemental Figure 2. Assessment of antibody specificity for detection of ACE2 by flow cytometry. Representative histograms using each of 13 different commercial ACE2 antibodies (summarized in Figure 1C and Table 1), comparing ACE2 staining of

ACE2-overexpressing HEK293T cells to ACE2 staining and secondary antibody only staining of parental cells.

A

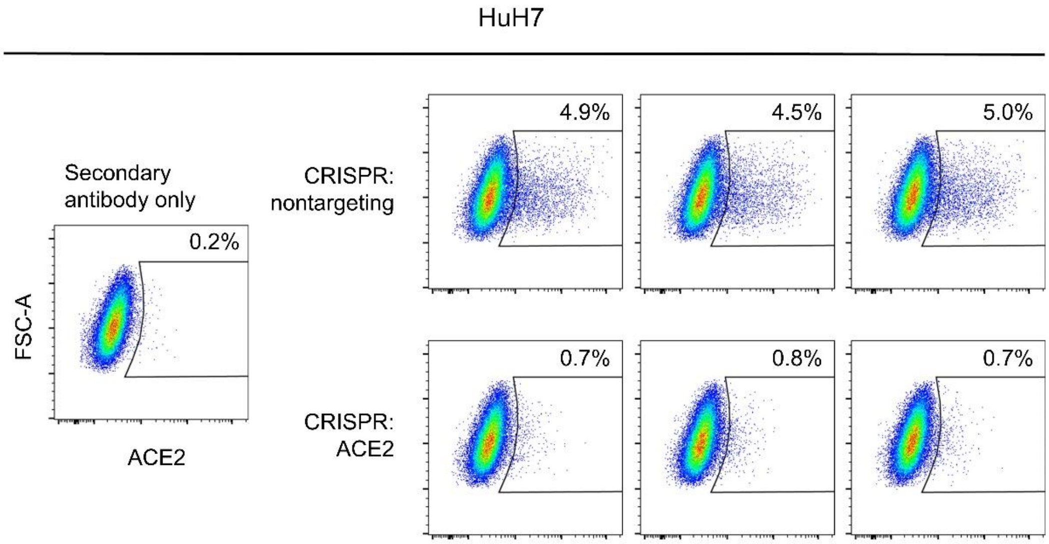

B

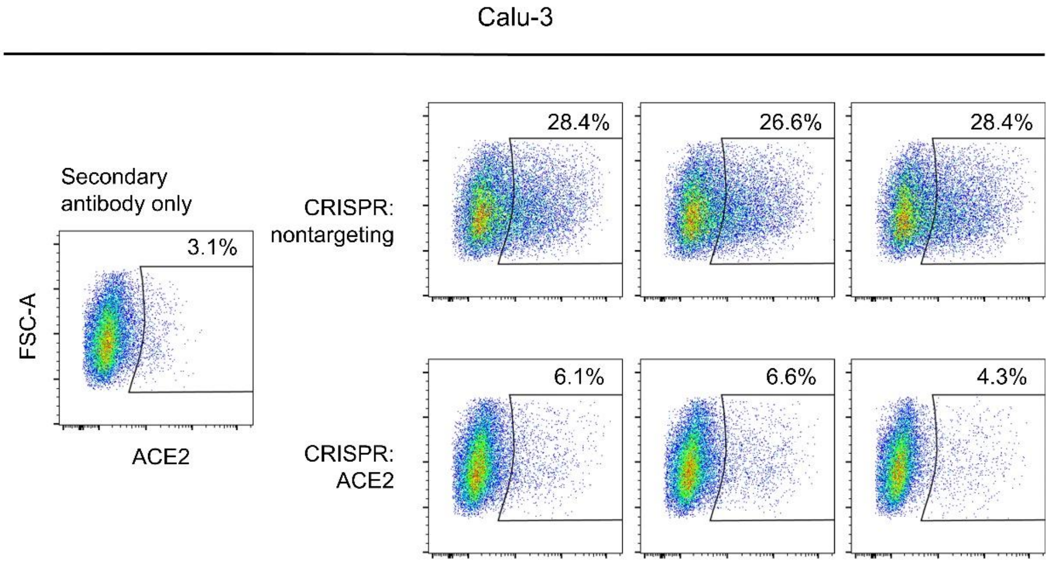

Supplemental Figure 3. Quantification of ACE2-positivity in HuH7 and Calu-3 cells by flow cytometry. Individual plots for three biological replicates of ACE2 staining by flow cytometry in HuH7 and Calu-3 cell lines generated by CRISPR with either a nontargeting guide (NT) or *ACE2*-targeting gRNA, (summarized in Figure 2C).

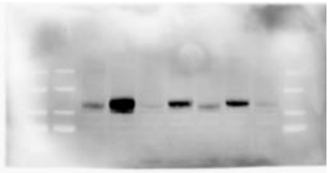

Figure 1A: ACE2

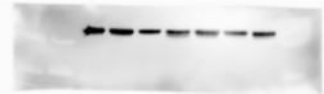

Figure 1A: β-actin

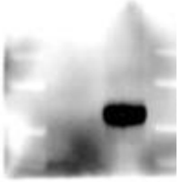

Figure 1B:
ACE2

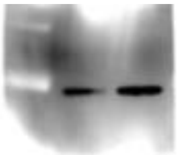

Figure 1B:
GAPDH

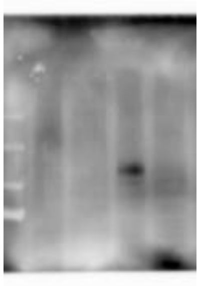

Figure 1D:
ACE2, Calu3

Figure 1D:
β-actin,
Calu3

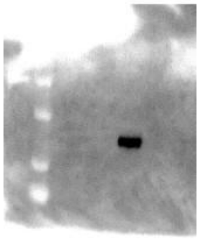

Figure 1D:
ACE2, HuH7

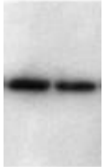

Figure 1D:
β-actin,
HuH7

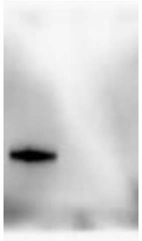

Figure 3C:
ACE2

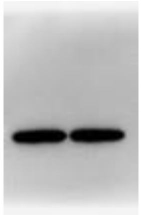

Figure 3C:
β-actin

Supplemental Figure 4. Full, unprocessed images of immunoblots corresponding to main text figures.
