# Supplementary material for: ACE2 protein expression within isogenic cell lines is heterogeneous and associated with distinct transcriptomes
Source: Sci Rep. 2021 Aug 5;11:15900. doi: 10.1038/s41598-021-95308-9 (PMC8342525; doi:10.1038/s41598-021-95308-9)
Supplement: Supplementary file 8 — Supplementary Legends. [file 41598_2021_95308_MOESM8_ESM.docx]

**Supplemental Table 1. Transcriptomes of ACE2-negative and ACE2-positive HuH7 cells.** Mapped reads for each transcript identified by RNA-seq of cells collected by flow cytometry sorting based on ACE2 surface abundance for each of 4 independent biologic replicates.

**Supplemental Table 2. Transcriptomes of parental wild-type and serially ACE2-enriched HuH7 cells.** Mapped reads for each transcript identified by RNA-seq of parental HuH7 cells and cells harvested after 3 rounds of serial ACE2 enrichment by flow cytometry for each of 3 independent biologic replicates.

**Supplemental Table 3. Differential gene expression analysis of sorted HuH7 ACE2-negative and ACE2-positive cells.** DESeq2 output of differentially expressed genes among RNA-seq profiles of cells collected by flow cytometry sorting based on ACE2 surface abundance for each of 4 independent biologic replicates.

**Supplemental Table 4. Differential gene expression analysis of HuH7 parental wild-type and serially ACE2-enriched cells.** DESeq2 output of differentially expressed genes among RNA-seq profiles of parental HuH7 cells and cells harvested after 3 rounds of serial ACE2 enrichment by flow cytometry for each of 3 independent biologic replicates.

**Supplemental Table 5. Hallmark gene set analysis of ACE2-correlated transcripts.** GSEA output with normalized enrichment score (NES), nominal p-value, and FDR for hallmark gene set analysis of either singly sorted ACE2-negative and ACE2-positive HuH7 cells, or wild-type and serially sorted ACE2-enriched HuH7 cells. Positive NES values indicate enrichment and negative values depletion in ACE2-expressing cells.

**Supplemental Table 6. Transcription factor target analysis of ACE2-correlated transcripts.** GSEA output with normalized enrichment score (NES), nominal p-value, and FDR for GTRD gene set analysis of either singly sorted ACE2-negative and ACE2-positive HuH7 cells, or wild-type and serially sorted ACE2-enriched HuH7 cells. Positive NES values indicate enrichment and negative values depletion in ACE2-expressing cells.
